# Lorazepam-Resistant Catatonia in an Antipsychotic-Naïve 24-Year-Old with Psychotic Symptoms

**DOI:** 10.1155/2020/2469707

**Published:** 2020-04-03

**Authors:** Juan Pablo Lucchelli, Stamatia Kourakou, Laia Pérez de Lucia Bové, Daniel Diaz Rodriguez

**Affiliations:** ^1^Hôpital du Jura Bernois, Pôle de Santé Mentale, L'Abbaye 22713 Bellelay, Switzerland; ^2^Laboratoire de Psychopathologie, E.A. 4050, Université de Rennes 2, France

## Abstract

Catatonia is a complex identifiable clinical syndrome characterized primarily by psychomotor symptoms. In recent decades, some authors have considered that catatonia can be presented as a catatonic syndrome in several pathologies such as bipolar disorder, schizophrenia and other psychotic disorders and not only in schizophrenia. Prior to DSM 5, there were two conceptions of catatonia: one in which clinical characterization seemed to play a determining role (a categorical view) and another in which a dimensional perspective advocated the existence of catatonia as a clinical entity in its own right, detached from the underlying pathology. Although there are no definitive consensus guidelines for the treatment of catatonia, some studies show that in the schizophrenic form of catatonia, benzodiazepines are partially effective, as well as treatment with ECT. We present the case of a 24-year-old man with severe catatonia and psychotic symptoms, resistant to lorazepam treatment, who achieved complete remission with clozapine treatment according to our diagnostic hypothesis of schizophrenia.

## 1. Introduction

Catatonia is an identifiable, complex clinical syndrome characterized mainly by psychomotor symptoms and, in the majority of cases, demanding inpatient treatment to avoid life-threatening complications. It was first described by Karl Kahlbaum in 1874, with many other researchers and clinicians coming to the same observation since then [1–3]. Although for the better part of the 20^th^ century catatonia was thought to be a subtype of schizophrenia and subsequently classified as such (from DSM I to DSM IV and ICD10) [4, 5], since a few decades ago, certain authors consider that catatonia is recognized to occur within a broad spectrum of psychiatric as well as medical illnesses. The most common psychiatric diagnoses that can be presented with a catatonic syndrome are bipolar disorder, unipolar depression, schizophrenia, and other psychotic disorders and autistic spectrum disorders [1–3, 6–8].

According to the DSM 5 [9], the diagnosis of catatonia (regardless of the underlying causative trouble) requires the presence of at least three of the following symptoms: catalepsy, waxy flexibility, mutism, stupor, agitation, negativism, mannerisms, posturing, stereotypies, grimacing, echolalia and echopraxia. That being said, mutism and stupor are the most common signs—each one present in over 90% of patients—and are considered the core criteria for the diagnosis [10–12]. In fact, two conceptions of catatonia arise in these last decades: one where a clinical characterization seems to play a determinant role (a categorical point of view, where the catatonic syndrome appears as an expression of a specific psychiatric or medical condition) and another one where a dimensional perspective takes an important place, advocating the existence of catatonia as a clinical entity of its own, completely detached from the underlying pathology. While the exact prevalence of the syndrome is unknown, some authors believe catatonia occurs in more than 10% of acute psychiatric inpatients [3, 6]. Fink [1] estimates that catatonia can have a broad range of diverse features, thus adopting a dimensional perspective, and considers that catatonia presentation is often subtle, and that is probably the reason why it is likely underdiagnosed by psychiatrists and other physicians [13]. Two separate subtypes have been described: the retarded and the excited types [12]. The first type of catatonia is characterized by the predominance of immobility, mutism, waxy flexibility, negativism, automatic obedience, staring, and posturing, while the excited type is associated with severe psychomotor agitation. Nonetheless, many patients present a clinical syndrome with combined symptoms of the two types.

An early diagnosis of the catatonic syndrome followed by an effective treatment is obviously of great importance, as the immobility and withdrawal from eating and drinking can result in numerous complications, such as malnutrition, dehydration, various infections, deep venous thrombosis and pulmonary embolism, pressure ulcers, or muscle contractures [12, 14]. Moreover, catatonia appears to be a risk factor for malignant neuroleptic syndrome, a severe condition with a high mortality rate.

Usually, catatonia's treatment consists of the administration of benzodiazepines (typically lorazepam in high doses) [10, 12]. Benzodiazepines are so widely established as a first-line treatment for this clinical syndrome that can be even used as a “diagnostic test” for catatonia, helping the clinician diagnose catatonia according to response to BZD treatment [6]. With a benzodiazepine treatment, a significant improvement of catatonic symptoms can be achieved in 70% of the cases (most of which have an affective component in the underlying pathology), while 30% need ECT or another type of pharmacological intervention.

Catatonia syndrome and its treatment among antipsychotic-naive patients presenting their first psychotic episode have not been widely studied. Valevski et al. [15] presented a 21-year-old psychotic patient with a catatonic status that responded well to a low-dose risperidone treatment. In 2010, Peralta et al. [5] assessed 200 first-episode, drug-naive psychotic patients for catatonia symptoms and found*n* = 24(12%) met the the Modified Rogers Scale criteria for a catatonic syndrome according to DSM IV. A one-month trial of treatment with haloperidol, olanzapine, or risperidone followed, with a significant improvement of catatonic symptoms of 20 out of 24 patients. This study showed no difference between FGA and SGA treatments, although the response was assessed dependently to the remission of the positive symptoms.

We are presenting a case report of a 24-year-old antipsychotic-naive patient with severe catatonia and psychotic symptoms, resistant to lorazepam treatment, of which we achieved complete remission with a clozapine treatment.

## 2. Case Report

A 24-year-old male is admitted to our clinic presenting a behavioral trouble described by his parents as a disorganized speech, disorientation, and memory deficits during the last few days. His medical examination at the ECU does not reveal any organic abnormalities, with the exception of a mild leukocytosis (total WBC count: 13.380) without an infection outbreak.

His medical history includes a meningoencephalitis of unknown origins at the age of 3 weeks and a diagnosis of an attention deficit hyperactivity disorder (ADHD) at the age of 9, treated with methylphenidate (40 mg per day). He experienced difficulties at school; he is described as having always been slightly intellectually inferior to his peers, with a hyperactive behavior. At the age of 20, after he got his certificate of commercial apprenticeship, the methylphenidate was stopped, but had to be reintroduced because of a “strange, impulsive behavior, marked by aggressiveness and excessive anger, where it was difficult for him to express himself,” according to his parents. He is not a smoker, nor does he use other psychotropic substances, and he is not known by the psychiatric services. He does not work, lives with his parents, and has a poor social life. He has never taken any antipsychotic treatment in his life.

Upon admission, we can observe a calm, distant, excessively cooperative patient with a nonspontaneous speech, with a prolonged latency in his answers and a poor production. Nonetheless, it is mostly comprehensible and coherent. The mood is labile, passing quickly from laughs to tears to anger, often inappropriate and out of context of the discussion. His thought process is characterized by poverty of production and a content marked by somatic delusions (that his organs do not work properly). He describes auditory hallucinations: different voices shouting at the same time or talking about him. The next day, he presents an episode of agitation and is withheld in therapeutic isolation.

After the first evaluation, given his clinical status, we stop the methylphenidate and introduce an antipsychotic treatment of olanzapine 20 mg/day, and given that after five days we do not get any positive evolution, we add amisulpride at 400 mg/day [16–18], as the psychotic symptoms and especially hallucinations are predominant in his clinical state. During the next few days, he develops rigidity, posturing, waxy flexibility, withdrawal from food and water, a repetitive speech, and automatic obedience. The Bush scale of catatonia score was 23, corresponding to a severe catatonia [19].

We diagnose a catatonic syndrome probably caused by a psychotic disease, and as a result, we discontinue all antipsychotic treatment. We reintroduce methylphenidate, as according to some studies it has a role in catatonia treatment and also lorazepam at 4 mg/day at the beginning, augmenting the dose until 10 mg/day, with a very poor response. We also schedule a brain MRI.

In the meantime, he is transferred to the ECU with an edema of the right leg with no pain or erythema and a body temperature of 37.8°C. A deep vein thrombosis and a right pneumonia are diagnosed, and he receives an antithrombotic and an antibiotic treatment. The brain MRI does not reveal any organic troubles. During his stay in the medical department, we gradually reduce the lorazepam treatment.

When he returns to the psychiatric department, after a thorough blood control and an ECG, we discontinue progressively lorazepam and introduce clozapine until the dose of 250 mg/day, following a classical titration, with a rapid and significant improvement of the catatonic symptoms after a few days. Soon, he eats and drinks normally, the motor disturbances disappear, and he is able to form spontaneous phrases from start to end. After two weeks, the CGI improvement scale (CGI-I) [20] is at two; after three weeks, it lowers to 1 (very much improved) [21]. We also add mirtazapine (until 30 mg/day) to potentialize the effects of the clozapine. The regular blood tests and ECGs show a good tolerance to the treatment. He is discharged with a treatment of clozapine, mirtazapine, and methylphenidate at the usual doses.

One month after discharge from hospital, our patient returns to his usual state: he told us that the auditory hallucinations were present since his adolescence and that they consisted of several voices talking among them or talking about him, so we can confirm the presence of the first rank of criteria for schizophrenia. The Bush catatonia rating scale score is down to 3/69.

## 3. Discussion

Catatonia is a complex syndrome synthesized by a constellation of symptoms, with both motor and speech abnormalities. The exact pathophysiological mechanisms remain to be defined; however, several neurobiological hypotheses have been proposed. Based on the pharmacologic properties of the currently indicated treatments, it is believed that catatonia is caused by a pathway dysregulation which implicates GABA-A, glutamate, and dopamine systems [8, 22–26]. More precisely, Neuhut et al. propose three pathophysiological hypotheses for the development of a catatonic state: lower binding of GABA-A in the right lateral orbitofrontal and parietal frontex, glutamate hyperactivity in the striatum, and dopamine hypoactivity in the prefrontal cortex [26, 27].

In many cases, in order for the underlying disease to be adequately assessed and diagnosed, acute catatonic symptoms must be effectively treated, while in others symptoms of the base illness coexist or even predominate, making it a challenge for the clinician to diagnose the catatonic syndrome.

Most of the patients with the syndrome respond rapidly to low-dose benzodiazepines, especially lorazepam in doses of 4-15 mg/day. Diazepam, oxazepate, and clonazepam have also been studied with similar effectiveness rates [6]. To this day, benzodiazepines remain the first-line treatment for catatonic patients, albeit significantly less effective in cases of schizophrenic catatonia compared to mood disorder-related catatonia (60% and over 90% response rates, respectively [2, 12]). Other GABA-A agonists such as zolpidem have also been studied with encouraging results [6] and even in order to make a diagnosis test [28]. In case of partial or nonresponse to lorazepam treatment, ECT appears to be the treatment of choice [7, 8] with a high efficacy, although also significantly lower among patients with schizophrenia diagnosis with respect to other pathologies [2].

The place of antipsychotics in the treatment of catatonia remains a subject of debate among researchers. It is generally advised to discontinue antipsychotic treatment upon suspicion of catatonia, as antipsychotic drugs (especially FGAs with a high anti-D2 potency) are believed to induce or aggravate catatonic symptoms. Nevertheless, there is increasing evidence supporting the use of atypical antipsychotics such as clozapine, olanzapine, risperidone, and quetiapine or partial dopamine agonists such as aripiprazole in the treatment of benzodiazepine-resistant catatonia with a psychotic substrate [25, 29–33]. More precisely, Naber et al. showed a high efficacy of clozapine among catatonic schizophrenic patients, with 90% of the sample presenting from slight amelioration to total remission [34]. Hung et al. presented two case reports with good response to clozapine, either to prevent catatonic episodes in the future or to relieve catatonic symptoms where the response to lorazepam ant to ECT was minimal [32]. Cassidy et al. reported a case of malignant catatonia that was treated successfully with high doses of olanzapine [31]. Martényi et al. showed a significant improvement of catatonic signs among 35 schizophrenic patients treated with olanzapine [35]. Valevski et al. [15] and Peralta et al. [5] showed a good response to risperidone use. Yoshimura et al. [36] reported a promising effect of quetiapine in treating schizophrenia with catatonic stupor in the acute phase, using a sample of 39 patients.

Other pharmacological agents, such as NMDA antagonists (amantadine, memantine) [37], antiepileptics (topiramate, carbamazepine) [38], stimulant agents like methylphenidate [39, 40], and antiserotonin agents [41] have been proposed by some researchers, though further research is required in order to establish their role in the pharmacological intervention for catatonic patients.

DSM 5 distinguishes the diagnostic of catatonia from the schizophrenia spectrum disorders, as it has been shown that it can accompany a plethora of psychiatric and medical illnesses. Nonetheless, along with other authors [2, 42, 43], we believe that the underlying disease should be taken highly into consideration when choosing the best therapeutic approach for each catatonic patient. That being said, schizophrenia and schizophrenic disorders occupy a particularly important place among those diseases, given that their diagnosis was inseparable from catatonia diagnosis up until a few decades ago. There is no doubt that different types of interventions are needed to treat psychosis, a bipolar disorder, an autistic spectrum disorder, and an autoimmune disease. Given that observation, a single treatment approach to treat all catatonic states regardless of the causative trouble would probably be erroneous.

This statement is supported by evidence that shows different response rates of benzodiazepine, ECT, or antipsychotic treatments depending on the underlying cause [2]. Catatonic schizophrenia is far less responsive to the established first-line treatment of lorazepam and ECT than, for example, catatonic mood disorders. Furthermore, a clinician should also consider the long-term therapeutic plan for their patients, in terms of efficacy, accessibility, and cost-effectiveness, once the catatonic state is successfully resolved.

In this case report, we were presented with a clinical status of both positive psychotic and catatonic symptoms. Although we posed ourselves the question of a possible catatonia case, we chose to treat the positive symptoms that, in this case, were predominant. Some days into the double antipsychotic treatment (amisulpride/olanzapine), the motor symptoms and the echophenomena were aggravated, possibly due to the first pharmacological intervention, and notably to amisulpride, due to its stronger antidopaminergic action. This evolution also served as an indicator for a clearer diagnostic hypothesis of catatonic syndrome. Thus, we diagnosed a catatonic schizophrenia and opted for a clozapine treatment for the catatonic symptoms, as well as the psychotic symptoms that were also very present and a cause of anxiety for the patient, leading us to the diagnostic hypothesis of schizophrenia. We observed encouraging results even from the second week of treatment which was well tolerated. Given his significant improvement, we decided that we are going to keep clozapine as the long-term antipsychotic treatment for this patient. Clozapine was chosen because of its highly atypical pharmacological structure and action mechanism. It is shown to have GABA-A-regulating potencies, as well as a serotonin-inhibiting action and a low affinity for D2 receptors, which can result in an upregulation of dopamine in the prefrontal cortex, eventually treating catatonic symptoms. Moreover, accumulating evidence shows a subtype of catatonia directly linked with clozapine withdrawal, revealing the probable therapeutic role of this molecule in treating a catatonic syndrome [44].

Another particular characteristic of the case presented is the ADHD diagnosis and the methylphenidate treatment. Methylphenidate has been recently studied as a potential therapeutic agent of catatonia, with a few case reports where it is successfully used to treat the syndrome, in the context of various underlying diseases [27, 39, 40]. The rationale for stimulant treatment in catatonia is provided by the “diminished dopamine hypothesis,” which proposed pathophysiological mechanism of the syndrome, according to which catatonia is caused by decreased dopamine activity in the mesostriatum [26, 27]. However, there are cases where we can observe catatonia-like symptoms after cocaine use [45], thus indicating a more complex etiological link. In this specific case, we have the coexistence of three diagnoses (ADHD, schizophrenia, and catatonia), each one of them implicating a different dopamine dysregulation pathway in its pathophysiological mechanism, making treatment choices a real conundrum. Moreover, in the patient's history, we have a failed attempt to stop the stimulant treatment 4 years ago because of the emergence of symptoms such as agitation, difficulties of speech, and impulsive behavior. There arises the question of a possible protective effect of methylphenidate for the patient over the years, given that both times its discontinuation caused an aggravation of his clinical state.

## 4. Conclusion

Despite the fact the dimensional point of view of catatonia brings an interesting perspective in the sense that we do not confine the diagnosis only to schizophrenia, clinicians should consider a prior psychiatric status and, in particular, schizophrenia, because response to treatment seems to be different according to the psychopathological original diagnosis. Further research is needed in order to clarify the precise pathophysiological pathway leading to this syndrome—common among so many psychiatric as well as nonpsychiatric diseases—so as to have more robust evidence concerning the optimal therapeutic approach for each patient group.
